# Assessment of Knowledge, Perception, and Practices Regarding Probiotics and Prebiotics Among Clinicians in Saudi Arabia: A Pilot Study

**DOI:** 10.7759/cureus.52080

**Published:** 2024-01-11

**Authors:** Noura M Eid, Ghadeer A Alsolami, Hadeel D Al-Nuafie, Haneen W Malibari, Wejdan D Alsolami, Sumia Enani

**Affiliations:** 1 Clinical Nutrition, Faculty of Applied Medical Sciences, King Abdulaziz University, Jeddah, SAU; 2 Nutrition, King Fahd Medical Research Center, King Abdulaziz University, Jeddah, SAU; 3 Food and Nutrition, Faculty of Human Sciences and Design, King Abdulaziz University, Jeddah, SAU

**Keywords:** dietitians, gastrointestinal physicians, irritable bowel syndrome, prebiotics, probiotics

## Abstract

Probiotics and prebiotics are important in preventing different diseases such as irritable bowel syndrome (IBS), which is a functional bowel disorder characterized by changes in bowel habits. Considering the limited studies on this topic, the present study was conducted to assess both gastroenterologist (GI) physicians’ and dietitians’ practices in recommending probiotics and prebiotics for IBS patients, as well as to measure their professional educational history on probiotics and prebiotics. A descriptive cross-sectional study was performed to investigate the knowledge, use, and perceptions of probiotics and prebiotics of GI physicians and dietitians at governmental hospitals in Jeddah, Saudi Arabia. A validated questionnaire was sent to 42 participants of both genders selected randomly, who were all qualified healthcare practitioners. The majority of GI physicians demonstrated high levels of knowledge about the health benefits of probiotics and prebiotics (76.2%). In contrast, most dietitians had lower levels of knowledge (52.4%), representing a significant difference between both groups (p<0.01). 83.3% of GI physicians believed that the use of probiotics and prebiotics was required for IBS patients, as compared with 50.0% of dietitians. In conclusion, GI physicians were shown to be more knowledgeable and believed more in the benefits of using probiotics and prebiotics in IBS patients than dietitians, but most participants were not aware of the probiotic products that are available in pharmacies in Saudi Arabia.

## Introduction

Irritable bowel syndrome (IBS), which affects approximately 11.2% of the population globally, represents a significant threat to public health for societies all over the world [1]. According to a study conducted by Canavan, West, and Card, approximately 30% of people who experience IBS symptoms will consult a physician [2]. A 2012 study conducted by Ibrahim et al. reported that 31.8% of the Saudi population complained of IBS. A 2022 study showed that the prevalence of IBS in Saudi Arabia has reached 18.2%, which was mostly associated with smoking, gastroesophageal reflux disease, stress, anxiety, and family history of IBS [3]. Recent studies have shown that IBS is associated with aging and poor quality of life (e.g., physical inactivity) [4]. Individuals with IBS may have different combinations of symptoms that interfere with their life. Abdominal pain or discomfort is the main characteristic of IBS; altered bowel movement is also common, including diarrhea, constipation, or both. IBS has different categories depending on stool consistency, which include IBS-D (diarrhea), IBS-C (constipation), and IBS-M (mix of both diarrhea and constipation). Prolonged contractions of the bowel may prevent the normal passage of air and cause bloating, belching, abdominal distension, and flatulence. Patients with IBS also complain of mucus in the stool and a feeling of incomplete evacuation. Some patients can control their symptoms by managing their diet, lifestyle, and stress. More severe symptoms can be treated by medication and counseling, such as establishing regular eating habits, eating small, frequent meals, drinking enough fluids, eating foods that are high in fiber, and using probiotics and prebiotics [5,6]. Research has shown that the consumption of prebiotics and probiotics has a strong impact on IBS [7].

The word probiotic is derived from Greek, meaning “for life’’ [8], with the term currently used for bacteria associated with beneficial effects for humans and animals [9]. The most recent definition of probiotic given by the International Scientific Association for Probiotics and Prebiotics (ISAPP) was “live microorganisms that, when administered in adequate amounts, confer a health benefit on the host” [10]. By contrast, the term prebiotic was initially introduced by Gibson and Roberfroid in 1995 who switched the “pro” prefix for “pre,” meaning “before” or “for,” defining a prebiotic as “a non-digestible food ingredient that beneficially affects the host by selectively stimulating the growth and/or activity of one or a limited number of bacteria in the colon” [9]. The most recent definition published in 2017 by the ISAPP includes non-carbohydrate substances [11].

Several clinical trials have proved that both prebiotics and probiotics have beneficial effects on IBS patients [12]. In a controlled clinical trial conducted in 2008, the consumption of prebiotics (3.5 g/day) of galactooligosaccharides (GOS) improved IBS symptoms and resulted in significant changes in stool consistency (p<0.05) and decreased flatulence [6]. In addition, both subjective global assessment (SGA) and anxiety scores (p<0.05) were improved in patients who received 7 g/day of prebiotics. In 2004, Tsuchiya conducted a single-blinded human study of 68 IBS patients to show the effect of probiotics, where there was a significant improvement in overall efficacy in 80% of patients at 12 weeks (p<0.01), with improvements to bloating, abdominal pain, and bowel habits [13]. Unfortunately, commercial probiotics and prebiotics are not widely available in Saudi Arabia. According to the Saudi Food and Drug Administration, there are only three approved products that contain probiotics and can be found in pharmacies. These are BIOGAIA probiotic drops, BION 3, and GLOVIT probiotic 60 chewable tablets. In 2017, Eid et al. carried out a descriptive study on the availability of prebiotics and probiotics in supermarkets, with the results showing a limited number and high cost of products [14]. By comparison, the United Arab Emirates has 37 probiotic and prebiotic products available in supermarkets [15].

The management of IBS can involve a collaboration between physicians and dietitians, where the role of gastroenterologists (GIs) is to diagnose and provide medical management. However, nutrition management such as the fermentable oligosaccharides, disaccharides, monosaccharides, and polyols (FODMAP) elimination diet has proven to be a first-line therapy [16]. Still, diet intervention may encounter challenges and other factors associated with patients’ immune status and gastrointestinal (GI) system, in which case recommending prebiotics and probiotics may be effective. There are several studies focused on assessing the knowledge of healthcare practitioners about probiotics and prebiotics. A previous study was carried out to assess physicians’ practice and their specific interest in GI disorders found that all physicians believed probiotics to be safe for most patients: 98% responded that probiotics have a role in treating GI symptoms, 93% have patients taking probiotics often for IBS, and most surveyed recommended probiotics for IBS patients [17]. Another study conducted in the UK found that probiotics are popular among GI physicians for the treatment of GI disorders [18].

Considering that there are no previous studies that have investigated the knowledge, use, and perceptions of probiotics and prebiotics among GI physicians and dietitians in Saudi Arabia, the current study is the first pilot study that aims to (1) translate and validate an English questionnaire to Arabic; (2) assess both GI physicians and dietitians on recommending probiotics and prebiotics for IBS patients in their clinical practices [19].

## Materials and methods

Study design and recruitment

This was a descriptive cross-sectional study on a total of 50 participants (dietitians and GI physicians) who were recruited from six governmental hospitals (King Abdul-Aziz University Hospital, East Jeddah Hospital, King Fahad Armed Forces Hospital, King Abdul-Aziz Medical City, King Fahad Hospital, and King Faisal Specialist Hospital) in Jeddah city, Saudi Arabia. Inclusion criteria included GI physicians and dietitians. Participants were recruited from the selected hospitals, where questionnaires were sent via department supervisors' emails and to all GI physicians and dietitians working in the same clinics. Sample size calculation was adapted from a previous study to provide a description of clinical dietitians' and physicians’ knowledge of probiotics and prebiotics, where only 50 filled questionnaires were completed [19].

Questionnaire translation and validation

A questionnaire was adapted from a previously published questionnaire [19]. Our questionnaire, which consisted of 15 questions, was developed, and validated by several steps to test the knowledge and practices of GI physicians and dietitians regarding probiotic and prebiotic treatments in clinical practice. The questionnaire is divided into three sections. The first section comprises demographic questions relating to age, gender, date of birth, field/area of practice, and contact information. The second section comprises questions designed to assess knowledge about probiotic and prebiotic treatment. The third section comprises information relating to beliefs and practices. The entire questionnaire was subject to all aspects of the validation process, which included a series of steps aimed at ensuring the validity and reliability of the questionnaire. The three steps are described below.

Step one was building the questionnaire, where content from relevant literature and research was transformed into statements and questions. In addition, the relation between the objectives of the study and their translation into content was established. The questionnaire aimed to measure the knowledge and practice of GI physicians and dietitians. This step focused on the following: writing statements and research questions; selection of appropriate scales of measurement; questionnaire layout; format, question order, and font size; front and back cover; and proposed data analysis. 

Step two was evaluating a draft of questions and content validity. The initial draft of the questionnaire contained 19 items across three sections: general information; knowledge about pro/prebiotics, and belief and practice. The items generated were then sent to an external expert group, which comprised three teaching dietitians with research experience who were expected to score the questionnaire based on their training, education, and exposure to nutrition. The purpose of this step was to evaluate each question in terms of its relevance, clarity, simplicity, and ambiguity by using a scale of 1 to 4, with a score of “4” used to reflect questions that were very relevant, very clear, very simple, and had a clear meaning, and a score of “1” used to reflect questions that were not relevant, not clear, not simple, and ambiguous. Each expert scored the questions in the item table. A mean score was then generated for each question within each category. Questions that scored ≤10 were reviewed and modified. Unsuitable questions were eliminated or changed if it was felt that they were not relevant or important for GI physicians and dietitians to know, or if the wording of the questions was unsuitable.

Step three was demonstrating pre-testing by cognitive interviewing, where the questionnaire was tested by cognitive interviews with two dietitians and two GI physicians to assess their comprehension of the questions. As a result, both the question's language and the overall layout of the questionnaire were improved.

Statistical analysis

Data were reported as frequencies and percentages (%) and analyzed using SPSS (version 21; IBM Corp., Armonk, NY). Differences between practice groups were evaluated by Pearson’s chi-squared test, where p<0.05 (two-sided test) was considered statistically significant.

Ethical approval

This study was approved by the research ethics committee of King Abdul-Aziz University (FAMS-EC2023-03). Participants signed a consent form to participate in the study and were informed that all data obtained from them would be kept confidential using codes instead of personal identifiers.

## Results

Descriptive data of participants 

As seen in Table 1, our sample size consisted of 50 physicians and 50 dietitians, most of whom were female and 20-29 years of age.

**Table 1:** Descriptive data of participants n is the number of participants; N% is the percentage of participants; GI=gastroenterologist.

Participants characteristics	n	N%
Gender		
Male	15	35.7%
Female	27	64.3%
Age category (years)		
20–29	18	51.4%
30–39	12	34.3%
≥40	5	14.3%
Practice		
GI physician	21	50.0%
Dietitian	21	50.0%

Participant's knowledge of probiotics and prebiotics 

As seen in Table 2, most of the study participants demonstrated high scores regarding their knowledge about probiotics and prebiotics definitions, as well as about probiotic strains and prebiotic types (73.8%, 59.5%, 61.9%, and 47.6%, respectively). As seen in Table 3, the majority of GI physicians had strong knowledge about the health benefits of probiotics and prebiotics (76.2%), whereas most dietitians had low knowledge of these benefits (52.4%), with a significant difference between these groups (ꭕ2=10.615, p<0.005). 83.3% of GI physicians believed that using probiotic and prebiotics was necessary for IBS patients, as compared with 50.0% of dietitians (ꭕ2=3.548, p=0.06).

**Table 2:** Knowledge of probiotics and prebiotics n is number of participants; N% is the percentage of participants; IBS-irritable bowel syndrome; IBD=irritable bowel disease; AAD=antibiotic-associated diarrhea

Knowledge	n	N%
Knowledge of probiotics definition		
No knowledge	11	26.2%
Knowledge	31	73.8%
Knowledge of prebiotics definition		
No knowledge	17	40.5%
Knowledge	25	59.5%
Knowledge of probiotics and prebiotics health benefits		
No knowledge	3	7.1%
Low knowledge	13	31.0%
High knowledge	26	61.9%
Knowledge of probiotic strains		
No knowledge	9	21.4%
Low knowledge	7	16.7%
High knowledge	26	61.9%
Knowledge of prebiotic types		
No knowledge	18	42.9%
Low knowledge	4	9.5%
High knowledge	20	47.6%
Knowledge of conditions that require using of probiotics and prebiotics		
No	4	9.5%
Yes	38	90.5%
IBS	25	65.8%
IBD	18	47.4%
AAD	27	71.10%

**Table 3:** Difference in knowledge between gastrointestinal physicians and dietitians n is number of participants; N% is the percentage of participants p>0.05, * p<0.05, ** p<0.01, *** p<0.001 IBS=irritable bowel syndrome; IBD=irritable bowel disease; GI=gastroenterologist; AAD=antibiotic-associated diarrhea

Knowledge	GI physicians		Dietitians		ꭕ2	P-value
	n	N%	n	N%		
Knowledge of probiotics definition						
No knowledge	5	23.8%	6	28.6%		
Knowledge	16	76.2%	15	71.4%	0.123	0.726
Total	21	100%	21	100%		
Knowledge of prebiotics definition						
No knowledge	8	38.1%	9	42.9%		
Knowledge	13	61.9%	12	57.1%	0.099	0.753
Total	21	100%	21	100%		
Knowledge of probiotics and prebiotics health benefits						
No knowledge	3	14.3%	0	0.0%		
Low knowledge	2	9.5%	11	52.4%	10.615	0.005
High knowledge	16	76.2%	10	47.6%		
Total	21	100%	21	100%		
Knowledge of probiotic strains						
No knowledge	5	23.8%	4	19.0%		
Low knowledge	4	19.0%	3	14.3%	0.408	0.816
High knowledge	12	57.1%	14	66.7%		
Total	21	100%	21	100%		
Knowledge of prebiotic types						
No knowledge	10	47.6%	8	38.1%		
Low knowledge	0	0.0%	4	19.0%	4.422	0.110
High knowledge	11	52.4%	9	42.9%		
Total	21	100%	21	100%		
Knowledge of conditions that require using of probiotics and prebiotics						
No	3	14.3%	1	4.8%		
Yes	18	85.7%	20	95.2%	1.105	0.293
Total	21	100%	21	100%		
If yes, for which conditions?						
IBS	15	83.3%	10	50.0%	3.548	0.060
IBD	7	38.9%	11	55.0%	1.293	0.256
AAD	9	50.0%	18	90.0%	8.313	0.004
Total	18	100%	20	100%		

Belief and practice about probiotics and prebiotics

As seen in Table 5, an equal proportion of GI physicians and dietitians believed that probiotics can be used in practice (90.5%). However, most GI physicians believed that probiotics can be used as supplements only (68.4%), whereas the majority of dietitians believe that probiotics can be used as both supplements and food (84.2%), with a significant difference between these groups (ꭕ2=13.612, p<0.001). Most of the subjects were not aware of probiotic products (80.5%), with no significant difference observed between the two groups (Table 4). 42.9% of the physicians and dietitians believed that probiotics are safe and effective in the treatment of certain GI-related illnesses or symptoms, with no significant difference between the two groups (Table 5). Most subjects did not have patients who took probiotics (59.5%); however, physicians and dietitians recommended probiotics and prebiotics treatment for IBS patients (66.7%), with no difference between the two groups (Table 5). 52.4% of subjects reported that when they do not recommend probiotics it is because there is currently a lack of familiarity on using probiotics in medical nutrition therapy in Saudi Arabia, where prescribed clinical medications are commonly used and controlled diet (Table 5).

**Table 4:** Belief and practice among physicians and dietitians n is number of participants; N% is the percentage of participants IBS=irritable bowel syndrome; UC=ulcerative colitis; CD=celiac disease; AAD=antibiotic-associated diarrhea

Belief and practice	n	N%
Using probiotics		
No	4	9.5%
Yes	38	90.5%
If yes, how?		
Supplement	15	39.5%
Food	1	2.6%
Both	22	57.9%
Awareness of probiotic products		
No	33	80.5%
Yes	8	19.5%
Believe that probiotics are safe		
Strongly disagree	0	0.0%
Disagree	0	0.0%
Undecided/Neutral	9	22.0%
Agree	21	51.2%
Strongly agree	11	26.8%
Believe that probiotics are effective in the treatment of certain GI-related illnesses or symptoms		
Strongly disagree	0	0.0%
Disagree	0	0.0%
Undecided/Neutral	12	28.6%
Agree	18	42.9%
Strongly agree	12	28.6%
Do you recommend any of probiotics and prebiotics, high-fiber diet, or short-chain fatty acids for IBS patients?		
No	7	17.9%
Yes	32	82.1%
If yes, how?		
Food	4	44.4%
Supplement	3	33.3%
Both	2	22.2%
What food/supplement do you usually recommend for IBS patients?		
Probiotic and prebiotic treatment	18	66.7%
High-fiber diet	12	44.4%
Short-chain fatty acids	1	3.7%
Do you have any patients who take probiotics?		
I don’t know	5	11.9%
No	25	59.5%
Yes	12	28.6%
If yes, how?		
Food	4	44.4%
Supplement	3	33.3%
Both	2	22.2%
For which condition?		
IBS	6	66.7%
UC	0	0.0%
CD	2	22.2%
AAD	6	66.7%
If you do not recommend, why?		
Currently lack of familiarity with the literature on probiotics.	11	52.4%
Feel there is not enough evidence to support their routine use in clinical practice.	7	33.3%
Believe the efficacy of probiotics to treat gastrointestinal symptoms is inferior to, or does not provide additional benefit over, standard therapeutics.	0	0.0%
All of the above	5	23.8%

**Table 5:** Differences in belief and practice among physicians and dietitians n is number of participants; N% is the percentage of participants p>0.05, * p<0.05, ** p<0.01, *** p<0.001 GI=gastroenterologist; IBS=irritable bowel syndrome; UC=ulcerative colitis; CD=celiac disease; AAD=antibiotic-associated diarrhea

Belief and practice	GI physicians		Dietitians		ꭕ2	P-value
	n	N%	n	N%		
Using probiotics						
No	2	9.5%	2	9.5%	0.000	1.000
Yes	19	90.5%	19	90.5%		
Total	21	100.0%	21	100.0%		
If yes, how?						
Supplement	13	68.4%	2	10.5%	13.612	0.001
Food	0	0.0%	1	5.3%		
Both	6	31.6%	16	84.2%		
Total	19	100.0%	19	100.0%		
Awareness of probiotic products						
No	15	75.0%	18	85.7%	0.749	0.387
Yes	5	25.0%	3	14.3%		
Total	20	100.0%	21	100.0%		
Believe that probiotics are safe						
Strongly disagree	0	0.0%	0	0.0%	0.953	0.621
Disagree	0	0.0%	0	0.0%		
Undecided/Neutral	4	19.0%	5	25.0%		
Agree	10	47.6%	11	55.0%		
Strongly agree	7	33.3%	4	20.0%		
Total	21	100.0%	20	100.0%		
Believe that probiotics are effective in the treatment of certain GI-related illnesses or symptoms						
Strongly disagree	0	0.0%	0	0.0%	5.000	0.082
Disagree	0	0.0%	0	0.0%		
Undecided/Neutral	9	42.9%	3	14.3%		
Agree	6	28.6%	12	57.1%		
Strongly agree	6	28.6%	6	28.6%		
Total	21	100.0%	21	100.0%		
Do you recommend any of probiotics and prebiotics, high-fiber diet or short-chain fatty acids for IBS patients?						
No	4	19.0%	3	16.7%	0.037	0.847
Yes	17	81.0%	15	83.3%		
Total	21	100.0%	18	100.0%		
If yes, which treatment?						
Probiotics and prebiotics treatment	9	64.3%	9	69.2%	0.537	0.464
High-fiber diet	8	57.1%	4	30.8%	0.875	0.350
Short-chain fatty acids	1	7.1%	0	0.0%	0.813	0.367
Total	14	100.0%	13	100.0%		
Do you have any patients who take probiotics?						
Yes	7	33.3%	5	23.8%	3.133	0.209
No	10	47.6%	15	71.4%		
I don’t know	4	19.0%	1	4.8%		
Total	21	100.0%	21	100.0%		
If yes, how?						
Food	0	0.0%	4	100.0%	9.000	0.003
Supplement	3	60.0%	0	0.0%		
Both	2	40.0%	0	0.0%		
Total	5	100.0%	4	100.0%		
If yes, for which condition?						
IBS	5	100.0%	1	25.0%	5.625	0.018
CD	1	20.0%	1	25.0%	0.032	0.858
UC	0	0.0%	0	0.0%	.	.
AAD	2	40.0%	4	100.0%	3.600	0.058
Total	5	100.0%	4	100.0%		
If you do not recommend why?						
Currently lack of familiarity with the literature on probiotics.	8	57.1%	3	42.9%	0.210	0.647
Feel there is not enough evidence to support their routine use in clinical practice.	5	35.7%	2	28.6%	0.050	0.823
Believe the efficacy of probiotics to treat gastrointestinal symptoms is inferior to, or does not provide additional benefit over, standard therapeutics.	0	0.0%	0	0.0%	.	.
All of the above	3	21.4%	2	28.6%	0.200	0.655
Total	14	100.0%	7	100.0%		

## Discussion

This paper demonstrates steps in translation and validation of a questionnaire that was used previously to assess clinicians' knowledge, perceptions, and practices using probiotics and prebiotics in the year [19]. Previous results have shown that participants were more familiar with probiotics (88%) than prebiotics (22%) and were motivated to start recommending both probiotics and prebiotics to their clients following strong evidence. However, regardless of their willingness to use prebiotics and probiotics with their patients, they were still hesitant and selective in choosing the types that had promising effects in the literature. On the other hand, in the present study, we demonstrated two groups of participants to show the role of dietetics in comparison with GI physicians, and data showed that physicians had more knowledge of using probiotics and prebiotics in comparison to dietitians. This difference may be due to the high involvement of physicians with gastrointestinal complications, who encounter the use of functional foods in comparison with dietetic practice. In agreement with the present study, in 2022, Alkhater conducted a study using a questionnaire to assess physicians’ understanding of the role of microbes in allergies, with the analysis indicating high scores related to their knowledge and awareness [20]. However, only 17.7% of the health workers had prescribed probiotics for their patients, and 37.8% were not aware of the reasons to prescribe such foods. Hence, there remains a lack of studies published regarding dietitians’ knowledge and use of probiotics and prebiotics in Saudi Arabia.

Our results showed that GI physicians were aware of the use of probiotics, especially in IBS patients, whereas clinical dietitians were aware of the use of probiotics especially in antibiotic-associated diarrhea. This difference may be due to the involvement of physicians with patients’ medical therapy, which lessens the role of dietitians. Other studies have assessed the use of probiotics in-depth, where practitioners not only differ in knowledge but also in practice. An international survey in 2019 showed that 90% of health practitioners thought that probiotics are beneficial in antibiotic therapy, where 70% believed they were beneficial for constipation, and a smaller percentage thought they were beneficial when traveling abroad [21]. In our study, more than half of GI physicians believed that probiotics could be used as supplements, whereas half of the dieticians believed that probiotics could be used as both supplements and food, indicating a significant difference between the two groups. Our study also showed that most participants were not aware of probiotic products, with no significant difference between practice groups. This result may be a consequence of the low availability of products in Saudi Arabia [14], as compared with other regions such as the United Arab Emirates [15].

Half of the participants reported that they do not recommend probiotics for two main reasons: First, they thought they were not familiar to be using probiotics in medical nutrition therapy; second, they felt that there was not enough evidence to support their routine use in clinical practice. This may be because of concerns related to safety and prolonged intake. A recent study in 2023 examining obstetricians’ awareness of probiotics, prebiotics, and microbiota confirmed that only 40% of participants believed that the intake of prebiotics and probiotics is safe for the patients. When compared with the results of other published studies, it should be noted that a study conducted in the USA showed that all GI physicians who participated believed probiotics to be safe for most patients, and 98% reported that probiotics have a role in treating gastrointestinal illnesses and symptoms. In addition, 93% of the GI physicians had patients taking probiotics most often for IBS. Most surveyed GI physicians recommended probiotics for IBS, antibiotic treatment, and Clostridium difficile-associated diarrhea because they believed that the literature supports using them for these conditions [17]. Our results were in contrast with those of other studies conducted in the USA, Europe, and Asia [19], which reported a high degree of familiarity with the term probiotics among healthcare providers. This difference may be a result of probiotic foods being reasonably well established in the USA, Europe, and Asia.

The current study is the first pilot study that validated an Arabic questionnaire to assess the knowledge and practice of both physicians and dietitians in Saudi Arabia. Thus, the study has several limitations, such as a low number of filled surveys, where only governmental hospitals have been targeted to ensure the practice is reprinted at the national level. According to the statistical results of this study, approximately 80.5% of the participants were not aware of the probiotic products that are available in pharmacies. Therefore, at the community level, there is a need for awareness campaigns about the availability of probiotic products in Saudi Arabia. In addition, there is a need for educational materials that include strong evidence about the benefits of using probiotics and prebiotics. Furthermore, at the hospital level, dietitians should be given more authority to prescribe these supplements. Yet, the validated survey will be used in future studies to cover all areas of Saudi Arabia including private sectors and clinics as well.

## Conclusions

Our study has successfully developed a useful survey to examine approaches used in medical nutrition therapy for GI health-related issues. Results of the current pilot survey study have shown that physicians had more knowledge and awareness of the benefits and use of probiotics and prebiotics compared to dietitians. 83.3% of GI physicians believed that using probiotics and prebiotics is required for IBS patients, compared to 50.0% of dietitians. This study could potentially be used as a basis for a larger study to determine the best way to reach healthcare providers and share the current research regarding the use of probiotics and prebiotics, as the participants were largely unaware of the availability of such products in Saudi Arabia. We suggest applying the survey on the national level to be able to use such data in improving curricula in health programs and ensuring the existence of clinical training on using probiotics and prebiotics in combination with medical therapy.
